# Ultra-High Cycling Stability of 3D Flower-like Ce(COOH)_3_ for Supercapacitor Electrode via a Facile and Scalable Strategy

**DOI:** 10.3390/molecules28196806

**Published:** 2023-09-26

**Authors:** Qing He, Wanglong Wang, Ning Yang, Wenmiao Chen, Xing Yang, Xing Fang, Yuanxiang Zhang

**Affiliations:** 1 Key Laboratory of Air-Driven Equipment Technology of Zhejiang Province, Quzhou University, Quzhou 324000, China; yang7092481@163.com (X.Y.); 36092@qzc.edu.cn (X.F.); 2 Department of Mechanical Engineering, Zhejiang University of Technology, Hangzhou 310058, China; 221122020226@zjut.edu.cn (W.W.); 15714178548@163.com (W.C.); 3 Department of Materials Science and Engineering, Tianjin University of Technology, Tianjin 300384, China; 20200988yangning@stud.tjut.edu.cn

**Keywords:** Ce(COOH)_3_, ultra-high, cycling stability, microwave-assisted, supercapacitor

## Abstract

An electrode material with high performance, long durability, and low cost for supercapacitors has long been desired in academia and industry. Among all the factors that affect the electrochemical performance and cycling stability of electrode materials, the morphology and intrinsic structure characteristics are the most important. In this study, a novel 3D flower-like Ce(COOH)_3_ electrode material was designed by taking advantage of the Ce^3+^ and -COOH groups and fabricated by a one-pot microwave-assisted method. The morphology and structure characteristics of the sample were examined by SEM, EDS, TEM, XRD, FT-IR, XPS, N_2_ adsorption/desorption techniques, and the electrochemical behaviors were investigated in a three-electrode configuration. The Ce(COOH)_3_ electrode presents an excellent specific capacitance of 140 F g^−1^ at 1 A g^−1^, higher than many other previously reported Ce-based electrodes. In addition, it delivers high rate capability that retains 60% of its initial capacitance when the current density is magnified 20 times. Dramatically, the Ce(COOH)_3_ electrode exhibits an ultra-high cycling stability with capacitance retention of 107.9% after 60,000 cycles, which is the highest durability among reported Ce–organic compound electrodes to the best of our knowledge. The excellent electrochemical performance is ascribed to its intrinsic crystal structure and unique morphology. This work indicates that the 3D flower-like Ce(COOH)_3_ has significant potential for supercapacitor applications and the facile and scalable synthesis strategy can be extended to produce other metal–organic composite electrodes.

## 1. Introduction

Global climate change, environmental pollution, and energy shortages have triggered the rapid development of renewable clean energy, including solar, wind, tidal, hydrogen, and biomass energy [1,2,3]. However, due to their high volatility, they are usually unable to be directly integrated into the power grid, and, therefore, advanced energy storage devices are urgently needed to provide more flexibility and reliability for taking advantage of new energy. Among various energy storage technologies, supercapacitors have attracted significant attention from academia and industry over the past few decades due to their distinguished properties, including much higher energy density than traditional capacitors and superior power density than batteries [4]. In addition, supercapacitors have also shown great potential in many other applications, such as aerospace, electric vehicles, microsensors, and portable electronics [5,6,7].

Supercapacitors (SCs) are typically composed of two electrodes separated by an electrolyte and can be classified into two main categories: electrochemical double-layer capacitors (EDLCs) and pseudocapacitors. EDLCs store energy by the adsorption of ions at the electrode/electrolyte interface, while pseudocapacitors store energy through fast and reversible faradaic redox reactions [8,9]. As a key component of supercapacitors, electrodes are usually fabricated with carbon-based materials, conductive polymers, transition metal oxides/hydroxides/sulfides/selenides/phosphates, and MXenes, which have their own advantages and disadvantages in terms of energy density, power density, and cycling stability [10,11,12,13]. Owing to their diverse topological architectures, optimizable synthesis protocols, adjustable porous structure, and abundant pseudocapacitor redox sites, metal–organic compounds have been explored as important candidates for supercapacitor applications. Cheng et al. summarized the latest reports on the first transition metal series of metal–organic compounds as electrode materials for supercapacitors, and it was found that the electrochemical performance of some metal–organic compounds had surpassed that of traditional electrode materials mentioned above [14]. Liu et al. concluded the most recent developments in metal–organic frameworks (MOFs)-based materials for SCs and highlighted the crucial effects of synthesis strategies on the sizes and morphologies of these materials. It is noted that the MOF compounds showed tremendous potential and significant advantages in spite of some existing challenges [15].

The main challenges for the application of metal–organic compounds come from their often insulating nature, limited cycling stability, and high cost. In recent years, the investigation of conductive metal–organic compounds has made inspiring progress. M.Dincǎ et al. obtained a highly conductive MOF, namely Ni_3_(HITP)_2_, through first principles calculations and experimental research, which possessed a bulk conductivity exceeding 5000 S m^−1^ [16]. Xu and Wang synthesized another type of conductive MOF, the Cu-CAT, which demonstrated a specific capacitance of 202 F g^−1^ at 0.5 A g^−1^. When the current density increased to 10 A g^−1^, it retained 66% of its initial capacitance [17]. Recently, Shen et al. investigated the node-to-node redox hopping behaviors of a Ce-MOF and coupled it with conductive CNTs to connect the charge-hopping pathways between different Ce-MOF nanocrystals, revealing that the issue of sluggish electronic conduction can be addressed [18]. However, there is not enough attention paid to improving the cyclic stability and reducing the preparation cost of metal–organic compounds. Most reported supercapacitors based on metal–organic compounds possess an electrochemical life that merely ranges from 1000 to 5000 cycles. In fact, the high stability of at least 10,000 cycles is the main requirement that distinguishes SCs from batteries. Therefore, it is urgently necessary to develop advanced SC electrodes with integrative performance to enable long cycling life and high capacity via facile and scalable strategies.

There are multiple factors that affect the cycling stability of electrodes based on metal–organic compounds, including the intrinsic properties of metal and organic ligands, the morphology, pore structure, etc. The strength of the coordination bond and the rigidity of the linker determine the thermodynamic stability and kinetic stability of metal organic compounds, respectively. Moreover, according to the hard and soft acid–base (HSAB) principle, high valent metal cations coupled with smaller radius have stronger coordination ability with hard base ligands, forming a more stable framework [19]. Han et al. prepared a trivalent metal MOF, namely Cr-MOF decorated with conductive polyaniline (PANI) and graphene oxide (GO), using a novel in situ polymerization method. The PANI/GO/Cr-MOF composite demonstrated high capacitance retention of 90.72% after 5000 cycles [20]. Yoon et al. synthesized a one-dimensional Ce(HPO_4_)_2_·xH_2_O electrode material via hydrothermal technique and it also delivered good long-term stability (92.7% after 5000 cycles) [21]. However, all the above-mentioned hydro/solvothermal methods or in situ chemical oxidative polymerization techniques are time-consuming and expensive, making it difficult to meet the needs of practical applications beyond labs. In recent years, microwave-assisted synthesis methods have attracted increasing attention by virtue of preparing materials rapidly, controllable, and with high yields [22,23,24,25,26]. Different from the traditional way of heating, the dielectric/volumetric heating mode of microwave-assisted methods is also preferable for synthesizing new materials with high purity and special microstructures. Younas et al. prepared nickel selenide (Ni_0.85_Se) nanosheets for supercapacitor electrodes using a single-step microwave-assisted method in just 10 min. The obtained Ni_0.85_Se electrode showed magnificent electrochemical properties [27]. Chen et al. synthesized a Zn-doped Ni-MOF by microwave-assisted method and hydrothermal method for comparison. The reaction time of the former was merely 6 min, much shorter than the 24 h of the latter. The sample by microwave-assisted method had a honeycomblike spherical morphology and the electrochemical performance was also much better [28]. 

Herein, in this work, a novel 3D flower-like Ce(COOH)_3_ was designed employing the high charge radius ratio Ce^3+^ ions and the well-known sturdy -COOH linkers. It was synthesized via a one-pot microwave-induced method, which provided two advantages. One thing is that the 3D flower-like Ce(COOH)_3_ exhibited a superior specific capacitance of 140 F g^−1^ at 1 A g^−1^, excellent rate capability, and ultra-high cycling stability at 10 A g^−1^ after 60,000 cycles (107.9% capacitance retention), which is one of the highest performances reported so far for Ce–organic compounds to our knowledge. The other is that the high purity and unique morphology of 3D flower-like Ce(COOH)_3_ could be synthesized rapidly with high yields. In fact, the microwave-assisted synthesis is so facile and efficient that it can significantly reduce the cost in scalable preparation of Ce(COOH)_3_ supercapacitor electrode material. The excellent electrochemical performance can be ascribed to the unique crystal structure and special morphology of the 3D flower-like Ce(COOH)_3_ and the facile and scalable synthesis strategy can be extended to other electrode materials based on metal–organic compounds.

## 2. Results and Discussion

**Figure 1:**
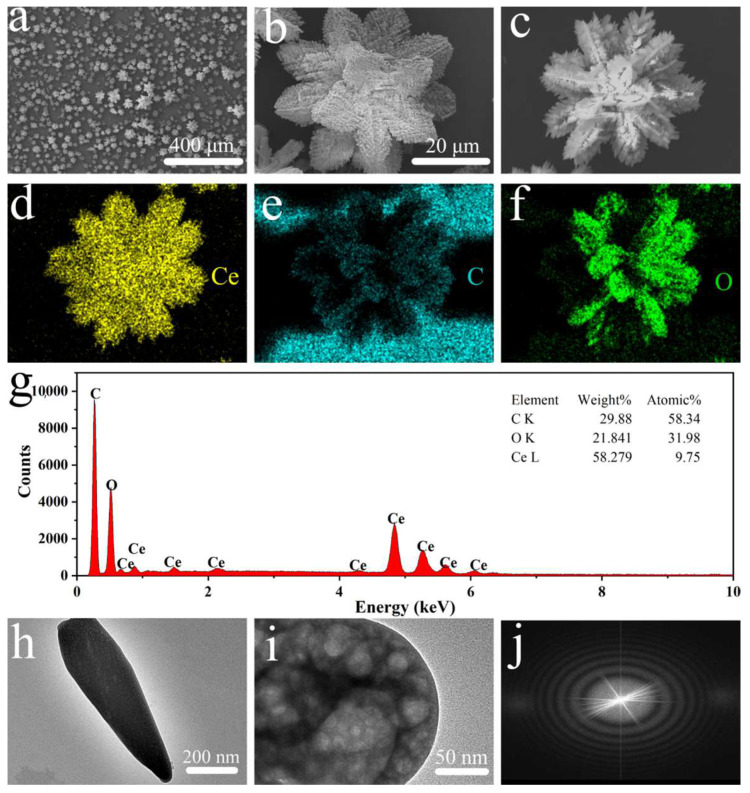
(**a**,**b**) SEM images of the 3D flower-like Ce(COOH)_3_. (**c**) The EDS images of Ce(COOH)_3_ with elemental mappings for (**d**) Ce; (**e**) C; (**f**) O; (**g**) EDS analysis of Ce(COOH)_3_. (**h**,**i**) TEM images of Ce(COOH)_3_ and (**j**) the corresponding selected-area electron diffraction (SAED) patterns.

The morphologies of the samples were observed by scanning electron microscopy (SEM). As shown in Figure 1a, the as-prepared Ce(COOH)_3_ particles are quite uniform and monodisperse, and the size of those ranges from 20 to 50 μm. A magnified SEM image in Figure 1b further reveals that the Ce(COOH)_3_ particle presents a beautiful three-dimensional (3D) flower-like shape, in which every petal of the flower is composed of numerous nanorods. EDS elemental mapping (Figure 1c–f) illustrates that Ce, C, and O were distributed throughout the prepared material. EDS analysis (Figure 1g) also verifies the element content of Ce, C, and O, which can preliminarily imply the production of Ce(COOH)_3_ successfully. TEM images have been taken to further verify the sub-units of the 3D flower-like Ce(COOH)_3_ structure. The magnified TEM image in Figure 1h provides the Ce(COOH)_3_ nanorods a mean diameter of 200 nm. The morphology and the size of the Ce(COOH)_3_ nanorods are in line with the details of the SEM images (Figure 1b,c). Moreover, it can be seen from Figure 1i that Ce(COOH)_3_ nanocrystals are fine and have a size of 10–30 nm and the SAED patterns show rings instead of spots, indicating the polycrystalline nature of the Ce(COOH)_3_ nanostructures (Figure 1j). 

**Figure 2:**
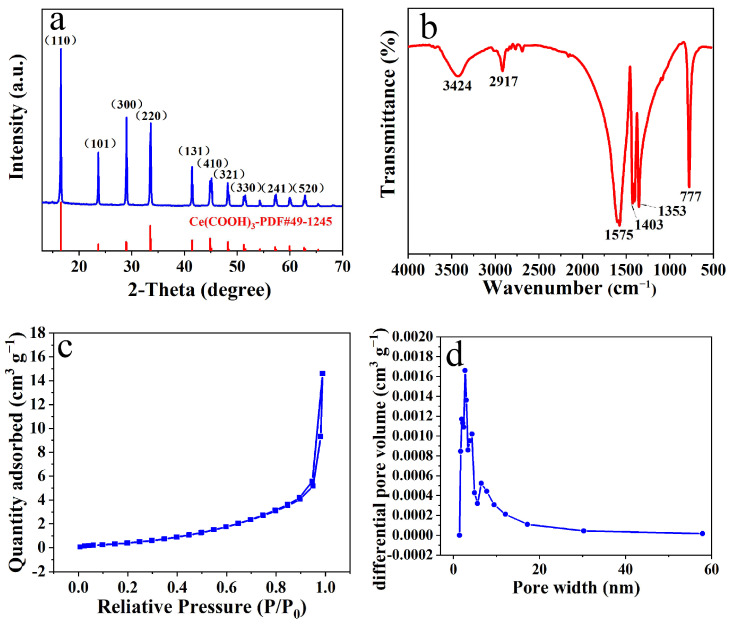
(**a**) X-ray diffraction (XRD) pattern, (**b**) Fourier transform infrared spectroscopy (FT-IR) spectrum, (**c**) N_2_ adsorption–desorption isotherm at 77 K, and (**d**) the corresponding BJH pore size distribution curve of the as-synthesized Ce(COOH)_3_.

In order to further confirm the formation of Ce(COOH)_3_ nanocrystalline, the as-prepared sample was examined by XRD and the patterns are demonstrated in Figure 2a. All the sharp Bragg peaks at 2θ values of 17°, 24°, 29°, 34°, 41°, 45°, 48°, 51°, 57°, and 63° can be indexed to (110), (101), (300), (220), (131), (410), (321), (330), (241), and (520) lattice planes of the rhombohedral phase of Ce(COOH)_3_ (PDF#49-1245), respectively. No other phase was detected, confirming the high purity of the products. The FT-IR spectrum of Ce(COOH)_3_ is shown in Figure 2b to better know its functional moieties. The broad absorption band at ~3424 cm^−1^ is assigned to the O–H stretching vibration of H_2_O, which could be related to the occurrence of some entrapped water. The absorption bands around 2917 cm^−1^ are attributed to C–H stretching vibrations. Also, two prominent bands arising at ~1575 cm^−1^ and ~1403 cm^−1^ are associated with the characteristics of the asymmetric and symmetric stretching vibrations of COO^-^, respectively. Moreover, the presence of a strong band at ~777 cm^−1^ is the feature of the stretching vibrational mode of the Ce–O bond.

Figure 2c,d exhibit the N_2_ adsorption/desorption isotherm and Barrett–Joyner–Halenda (BJH) pore size distribution curve of the as-synthetized Ce(COOH)_3_. As per the IUPAC classification, the isotherm of Ce(COOH)_3_ can be assigned to the Langmuir type IV isotherm. The N_2_ adsorption isotherm that slightly creeps up in the low relative pressure (P/P_0_) range indicates the existence of micropores, while the distinct hysteresis loop in the range of 0.8–1.0 P/P_0_ signifies the abundance of mesopores between the Ce(COOH)_3_ nanorods. As calculated from the desorption branch of the isotherm, the pore sizes of the Ce(COOH)_3_ range from 1.7 to 50 nm and mostly center around 2.7 nm. A rational hierarchical pore structure would facilitate electrolyte ion diffusion and buffer the material volume change during charge/discharge cycles, thereby proving beneficial for the electrochemical performance and cycling stability [29]. The specific surface area calculated by the Brunauer–Emmett–Teller (BET) method is 3.57 m^2^ g^−1^, indicating that the capacitance of Ce(COOH)_3_ mainly originates from pseudo-capacitance rather than the EDLC mechanism [30].

**Figure 3:**
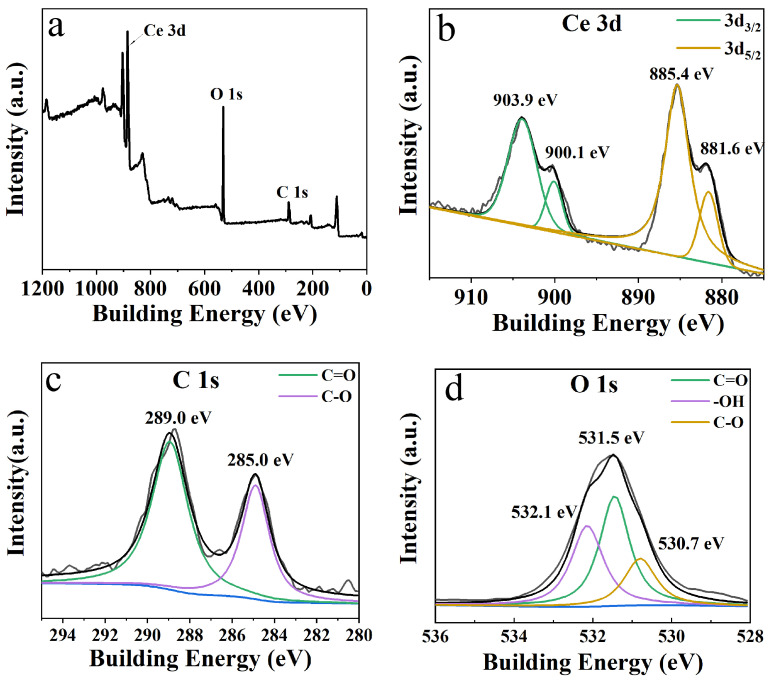
(**a**) X-ray photoelectron spectra (XPS) survey spectrum of the as-synthesized Ce(COOH)_3_ and high-resolution XPS scan of (**b**) Ce 3d, (**c**) O 1s, (**d**) C 1s binding energies.

X-ray photoelectron spectroscopy (XPS) was used to study the elemental content and valence states of the Ce(COOH)_3_ sample. Figure 3a shows the XPS survey spectrum, revealing the existence of Ce, O, and C. Figure 3b–d present the high-resolution XPS spectra of Ce 3d, O 1s, and C 1s, respectively. The high-resolution XPS spectrum of Ce 3d can be deconvoluted to four Gaussian peaks located at 881.6 eV, 900.1 eV (Ce 3d^9^ 4f^2^) and 885.4 eV, 903.9 eV (Ce 3d^9^ 4f^1^). The peak positions of Ce 3d_5/2_ (881.6 eV, 885.4 eV) and Ce 3d_3/2_ (900.1 eV, 903.9 eV) are consistent with the spin–orbit features of Ce^3+^. The scan spectrum of O 1s can be deconvoluted to three peaks at 530.7 eV, 531.5 eV, and 532.1 eV, belonging to C–O, C=O, and -OH, respectively. The characteristic peaks of C 1s centered at 285 eV and 289 eV are assigned to C-O and C=O, respectively. The above results thoroughly reveal the electronic spin states of each element in the 3D flower-like Ce(COOH)_3_ structure and are in good agreement with previous research reports [31,32,33]. 

**Figure 4:**
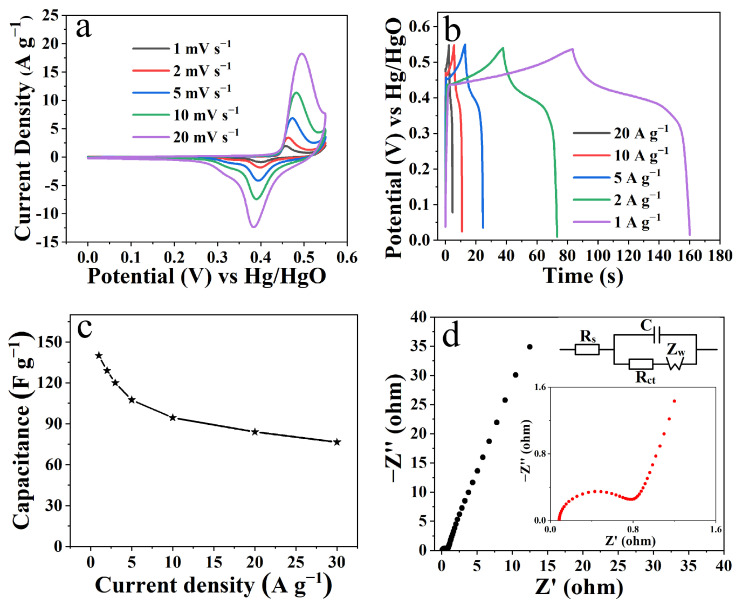
(**a**) CV curves of Ce(COOH)_3_ at various scan rates. (**b**) GCD curves of Ce(COOH)_3_ at different current densities. (**c**) The specific capacitances of Ce(COOH)_3_ calculated from GCD curves at different current densities. (**d**) Nyquist impedance spectrum of Ce(COOH)_3_.

The electrochemical properties of the 3D flower-like Ce(COOH)_3_ were investigated in a classic three-electrode system using 3 M KOH as the electrolyte, of which a Pt foil was used as the counter electrode, a commercial Hg/HgO electrode as the reference electrode, and the as-prepared Ce(COOH)_3_ as the working electrode. As shown in Figure 4a, the CV curves of the Ce(COOH)_3_ were collected at various scan rates (1–20 mV s^−1^) in the potential window from 0.0 to 0.55 V. It is clearly observed that all the CV curves deviate from the rectangular shape and present obvious reversible redox peaks, revealing the pseudo-capacitance properties of Ce(COOH)_3_. The anodic peaks and cathodic peaks center around +0.48 V and +0.38 V, respectively, corresponding to the Faradaic redox reaction of Ce^3+^/Ce^4+^ sites. Furthermore, as the scan rates increase, the redox current densities also increase while the profile of the CV curves is almost unchanged, indicating an outstanding rate capability and reversibility for energy storing. 

Figure 4b presents the galvanostatic charge–discharge (GCD) curves of the Ce(COOH)_3_ electrode under various charge–discharge current densities (1–20 A g^−1^). Despite the different current densities, all the GCD curves display similar symmetrical shapes with a certain deviation from an isosceles triangle, indicating the reversible faradaic redox reaction behavior of Ce(COOH)_3_, which is also in good agreement with the results of the CV curves. Then, the specific capacitances of Ce(COOH)_3_ at each current density are calculated using the GCD curves and the results are shown in Figure 4c. It can be seen that the Ce(COOH)_3_ electrode delivers high capacitances of 140, 129, 107.5, and 94.5 F g^−1^ at the current densities of 1, 2, 5, and 10 A g^−1^, respectively. Even when the current density is magnified to 20 A g^−1^, a capacity of 84 F g^−1^ is still retained (the capacitance retention rate is 60%, corresponding to the initial value at 1 A g^−1^), indicating its high rate capability. It is worth noting that the capacitive performance of Ce(COOH)_3_ holds a significant advantage among the previously reported Ce-based electrodes, such as Ce(OH)_3_ (75 F g^−1^ at 5 mV s^−1^) [34], graphene/CeO_2_ (89 F g^−1^ at 1 A g^−1^) [35], Ce-BTC (94.8 F g^−1^ at 1 A g^−1^) [36], Ce(HPO_4_)_2_.xH_2_O (114 F g^−1^ at 0.2 A g^−1^) [21], and carbon/CeO_2_-PVP (123.8 F g^−1^ at 0.25 A g^−1^) [37]. Such superior specific capacitance benefits from the unique crystal structure and special 3D flower-like morphology that offer incredibly rich Ce^3+^/Ce^4+^ active sites.

Furthermore, to study the dynamic transfer process and frequency response of the charge and electrolyte ions in the Ce(COOH)_3_ electrode, the electrochemical impedance spectroscopy (EIS) was measured. As depicted in Figure 4d, the corresponding Nyquist plot is obtained and the inset is the magnified part for the high-frequency region as well as the equivalent fitting circuit. The Nyquist plot consists of a quasi-semicircular part in the high-frequency range (charge transfer process) and an inclined linear part (diffusion-limited process) in the low-frequency range. In the high-frequency range, the intercept on the real (Z’) axis signifies the equivalent series resistance (R_s_), including the intrinsic resistance of the Ce(COOH)_3_ electrode material, the ohmic resistance of the KOH electrolyte, and the interfacial resistance between the active material and the Ni foam current collector. From the Nyquist curve, the R_s_ value is calculated to be 0.16 Ω, indicating the high conductivity of the Ce(COOH)_3_ electrode material. The low R_s_ is beneficial for the rate capability and avoiding unnecessary heat generation during the electrochemical working process. Then, the diameter of the quasi-semicircular part (or kinetic loop) in the Nyquist plot represents the charge transfer resistance (R_ct_), which mainly originates from the Faradaic reactions occurring at the interface between electrode and electrolyte and of course the persistent double-layer capacitance (C_dl_). According to the results of the equivalent circuit fitting, the R_ct_ of the Ce(COOH)_3_ electrode is quite low (0.63 Ω), implying a fast charge transfer at the electrode–electrolyte interface. In the mid-frequency range, the straight line with a slope of 45° is known as the Warburg line. The Warburg resistance (W_d_) reflected by the Warburg line comes from the frequency dependence of the ions’ diffusion behavior from electrolyte to electrode surface. Apparently, the short Warburg line of the Nyquist plot for the Ce(COOH)_3_ electrode denotes a smooth and unobstructed ion transport process, resulting in low W_d_. In the low-frequency range, the inclined linear part of the Nyquist plot represents the limit capacitance (C_L_) and provides signals on the ideality of the Ce(COOH)_3_ electrode towards supercapacitive behavior. The linear part more parallel to the imaginary (Z″) axis purports more ideal capacitive behavior. Obviously, the Ce(COOH)_3_ electrode demonstrates a nearly vertical line, indicating outstanding capacitive performance. Based on the above analysis, the Ce(COOH)_3_ electrode material possesses excellent impedance properties, which can also be attributed to its crystal structure and special morphology. The high crystallinity of the Ce(COOH)_3_ provides a highway for the electronic transfer, reducing the internal resistance of the material. The 3D flower-like hierarchical Ce(COOH)_3_ particles exhibit small and uniform size and consist of numerous nanorods orderly interconnecting each other, which can facilitate the migration of the electrolyte ions, shorten the diffusion paths to the electrode surface, and decrease the interfacial charge transfer resistance.

**Figure 5:**
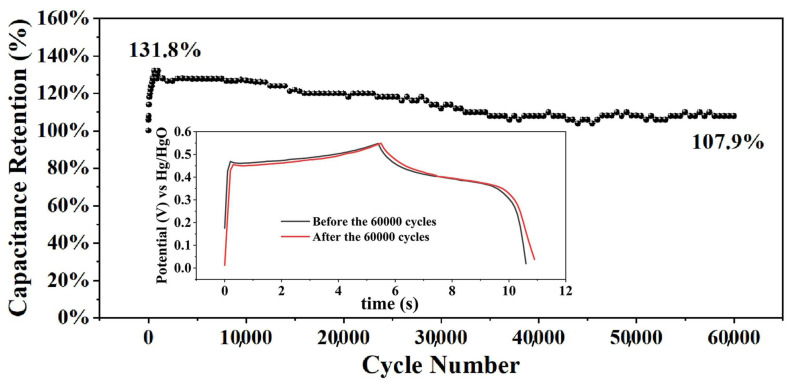
Cyclic performance of Ce(COOH)_3_ at 10 A g^−1^ for 60,000 cycles and the inset is the GCD curves of the first cycle and 60,000th cycle.

Most importantly, the electrochemical stability is a significant property of a supercapacitor electrode. Therefore, the Ce(COOH)_3_ electrode was subjected to a long-term cyclic test at the constant current density of 10 A g^−1^. As shown in Figure 5, the specific capacitance of the Ce(COOH)_3_ electrode retains 107.9% of its initial value after experiencing 60,000 cycles. At the same time, it can be seen from the inset of Figure 5 that there is no obvious change between the charge–discharge curves before and after the cycling test. This ultra-high cycling stability and remarkable reversibility of Ce(COOH)_3_ were first discovered in this work. In fact, as summarized in Table 1, the cycling stability and capacitance retention rate of Ce(COOH)_3_ are the highest among the reported Ce–organic compounds to the best of our knowledge [18,21,36,38,39,40,41,42]. Moreover, the cycling stability and capacitance retention rate of Ce(COOH)_3_ also have substantial advantages among the other most reported metal–organic compounds known for their long cycle life, such as Ni_3_(HITP)_2_ (90% after 10,000 cycles at 2 A g^−1^) [16], Co-MOF (96% after 10,000 cycles at 5 mA cm^−2^) [43], Ni/Co-MOF (89.8% after 12,000 cycles at 10 A g^−1^) [44], Cu_3_(HHTP)_2_ (79.9% after 5000 cycles at 5 A g^−1^) [45], V-MOF (92.1% after 10,000 cycles at 1 A g^−1^) [46], Cr-MOF (85% after 10,000 cycles at 0.5 A g^−1^) [47], Mn-MOF (81.18% after 10,000 cycles at 10 A g^−1^) [48], and Fe-MOF (74.4% after 10,000 cycles at 1 A g^−1^) [49].

The ultra-high cycling stability and remarkable reversibility of Ce(COOH)_3_ can be attributed to the following aspects. The primary one is the special 3D flower-like morphology. As can be seen in Figure 5, the capacitance of Ce(COOH)_3_ increases in the initial 2000 cycles because the stack-up Ce(COOH)_3_ petals may lead some Ce^3+^ to be in a deep position where the electrolyte cannot easily reach. In pace with the charge–discharge cycles, an increasing number of Ce^3+^ are activated and eventually result in a peak capacitance retention rate of 131.8%. After that, the capacitance retention value shows a slowly decreasing tendency and gradually stabilizes around 107.9% after 60,000 cycles. Similar phenomena regarding that the capacitive retention is over 100% have also been found in other researchers’ works [42,50]. Generally, an electrochemical electrode dominated by a pseudocapacitance mechanism may experience large volume change during charging and discharging, leading to disintegration or partial detachment and further rapid deterioration of capacitance and service life. However, the hierarchical 3D flower-like morphology provides generous free space to buffer the volume change and shortens the diffusion paths of ions by exposing internal active sites to the electrolyte directly, thereby ensuring ultra-high long-term cycling stability and capacitance retention rate. In addition, the role of material composition design and its crystal structure cannot be ignored. According to the hard and soft acid–base (HSAB) principle, Ce^3+^ coupled with high valent and small radius can bond firmly with -COOH groups. The good mechanical strength of Ce(COOH)_3_ can prevent it from pulverization during long-term charging and discharging. The rapid redox capability of the Ce^3+^/Ce^4+^ pair and enhanced charge transfer from the high crystallinity of Ce(COOH)_3_ also pave the way for its ultra-high cycling stability and extraordinary reversibility for energy storing.

## 3. Materials and Methods

### 3.1. Materials

Ammonium cerium(IV) nitrate (Ce(NH_4_)_2_(NO_3_)_6_) and formic acid (HCOOH) were purchased from Macklin chemical reagent Co., Ltd. (Shanghai, China). Ethylene glycol and ethyl alcohol were obtained from Xilong scientific Co., Ltd. (Shantou, China). Acetylene black and Polyvinylidene fluoride were purchased from Tianjin Chemical Technology Co., Ltd. (Tianjin, China). Potassium hydroxide (KOH) was acquired from Sinopharm Chemical Reagent Co., Ltd. (Shanghai, China). All the chemical reagents were analytical reagent (AR) and directly used without further purification.

### 3.2. Preparation of Ce(COOH)_3_

**Scheme 1:**
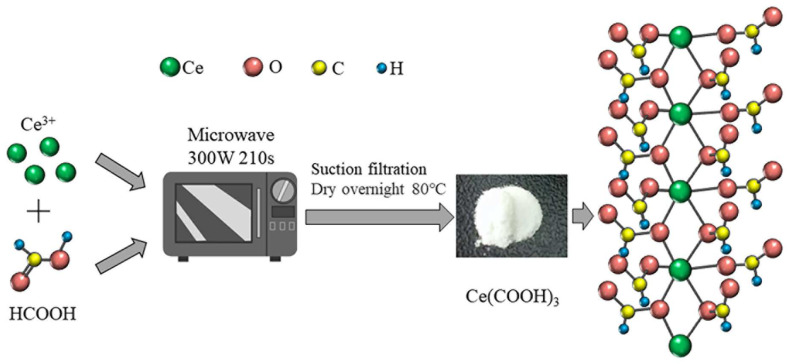
Schematic for the synthesis of Ce(COOH)_3_ using a one-pot microwave-assisted method.

The one-pot microwave-assisted synthesis process of Ce(COOH)_3_ is demonstrated in Scheme 1. In a typical run, 3.506 g Ce(NH_4_)_2_(NO_3_)_6_ was dissolved in 12 mL ethylene glycol and stirred for 30 min to form a homogeneous solution. After adding 41.2 mL HCOOH into the above solution and continuing stirring for 3 min, the mixture was transferred into a microwave reactor (Panasonic NN-GF352 M, 2450 MHz, Osaka, Japan) under 300 W for 210 s. Then, the obtained suspension was treated by vacuum filtration processing and the precipitate was washed with ethanol and deionized water alternately until the filtrate was neutral. Finally, the collected white powder was dried in vacuum at 80 °C for 12 h.

### 3.3. Characterization

The crystalline structure of the samples was characterized by X-ray diffraction (XRD, Rigaku Ultima IV, Tokyo, Japan) using Cu Kα radiation (*λ* = 1.5406 Å). Fourier transform infrared spectroscopy (FTIR) was recorded on a Fourier transform infrared spectrometer (Thermo Fisher Nicolet 6700, Waltham, MA, USA). The morphologies of the samples were observed by scanning electron microscopy (SEM, Hitachi SU-8100, Tokyo, Japan) attached with an energy dispersive X-ray spectrometer (EDS, IXRF3310, Tokyo, Japan) and transmission electron microscopy (TEM, FEI Tecnai G2 F30, Hillsboro, OR, USA). The specific surface area was collected by Brunauer–Emmett–Teller (BET) method using an Autosorb-iQ system (Quantachrome Instruments, Boynton Beach, FL, USA) operated at −196 °C. The content and configuration of C, O, and Ce were analyzed by X-ray photoelectron spectroscopy (XPS, Escalab 250XI, Waltham, MA, USA).

### 3.4. Electrochemical Measurement

The electrochemical performance of the samples was investigated using an electrochemical workstation (CHI 660E), including a classic three-electrode system. The working electrode was prepared by well grinding Ce(COOH)_3_ material, acetylene black, and polyvinylidene fluoride (PVDF) in ethanol to form a homogeneous slurry. Their mass ratio was 8:1:1. The as-prepared slurry was coated onto a Ni foam (1 × 1 cm^2^) and vacuum-dried at 80 °C overnight. Subsequently, it was pressed at 8 MPa to obtain the final working electrode. A typical mass loading of the active material was ~1 mg cm^−2^. A planar Pt foil and a commercial Hg/HgO electrode were used as counter and reference electrodes, respectively. The electrolyte was 3 M KOH solution. Cyclic voltammetry (CV) tests were performed in the potential range of 0–0.55 V at scan rates of 1–20 mV s^−1^. Galvanostatic charge–discharge (GCD) measurements were conducted from 0 to 0.55 V at the current densities of 1–20 A g^−1^. The Nyquist plots were collected via electrochemical impedance spectroscopy (EIS) at the open circuit potential with frequency ranging from 100 kHz to 1 Hz. The specific capacitances of the electrodes were calculated according to their GCD profiles using the following formula.

(1) $$C_{s}=\frac{i\times \Delta t}{\Delta V\times m}$$

where i is the constant current (A) in the charging and discharging process, Δt is the discharge time (s), ΔV  is the potential window (V), and m is the mass of active material on the electrode (g).

## 4. Conclusions

In this work, a novel 3D flower-like Ce(COOH)_3_ electrode material with ultra-high cycling stability was designed by taking advantage of the Ce^3+^ and -COOH groups according to the HSAB principle and fabricated by a one-pot microwave-assisted method. The obtained Ce(COOH)_3_ sample is well crystalline and delivers a high specific capacitance of 140 F g^−1^ at 1 A g^−1^, which is one of the highest capacitance values reported for Ce–organic compounds. In addition, the Ce(COOH)_3_ sample demonstrates high rate capability that retains 60% of its initial capacitance when the current density is magnified 20 times. Prominently, the Ce(COOH)_3_ exhibits ultra-high cycling stability with capacitance retention of 107.9% after 60,000 cycles, which is also the highest among the reported Ce–organic compound electrodes and far better than most of the other reported metal–organic compounds. The excellent electrochemical performance of Ce(COOH)_3_ can be attributed to its intrinsic composition and structural characteristics, including the rapid redox capability of Ce^3+^/Ce^4+^, the strong coordination bond, the high crystallinity, and, most importantly, the unique 3D flower-like morphology. These results indicate that Ce(COOH)_3_ has significant potential for supercapacitor applications and the facile and scalable one-pot microwave-assisted method can be explored to produce electrode materials with outstanding cycling stability, excellent electrochemical performance, and high efficiency.

## Figures and Tables

**Table 1:** The cycling stability comparison between the Ce(COOH)_3_ in this work and other metal–organic compounds reported for supercapacitors.

Sample	Cycles	Capacitive Retention	Ref
Ce-H_2_L	2000/4 *^a^*	64.0%	Ref [38]
Ce-MOF-808/CNT	2000/1 *^b^*	71.0%	Ref [18]
Ce-MOF-808/PEDOT	2000/0.5 *^b^*	90.0%	Ref [39]
Ce-BTC (rod-like)	4000/250 *^c^*	52.8%	Ref [40]
Ce-MOF	5000/4 *^a^*	65.2%	Ref [41]
Ce-BTC (nanorod)	5000/3 *^a^*	83.0%	Ref [36]
Ce(HPO_4_)_2_.xH_2_O	5000/3 *^a^*	92.7%	Ref [21]
Ce-UiO-66/TNF	10,000/50 *^b^*	95.0%	Ref [42]
Ni_3_(HITP)_2_	10,000/1 *^b^*	84.0%	Ref [16]
Co-MOF	10,000/5 *^b^*	96.0%	Ref [43]
Ni/Co-MOF	12,000/10 *^a^*	89.8%	Ref [44]
Cu_3_(HHTP)_2_	5000/5 *^a^*	79.9%	Ref [45]
V-MOF	10,000/1 *^a^*	92.1%	Ref [46]
Cr-MOF	10,000/0.5 *^a^*	85.0%	Ref [47]
Mn-MOF	10,000/10 *^a^*	81.18%	Ref [48]
Fe-MOF	10,000/1 *^a^*	74.4%	Ref [49]
Ce(COOH)_3_	60,000/10 *^a^*	107.9%	This work

*^a^* = A g^−1^; *^b^* = mA cm^−2^; *^c^* = mA s^−1^.

## Data Availability

Data are available upon reasonable request.

## Sample Availability

Samples are available upon reasonable request.

## References

[1] Turner J.M. (2022). The matter of a clean energy future. Science.

[2] Editorial (2023). Clean energy can fuel the future—And make the world healthier. Nature.

[3] Xia G. (2022). The Chemistry of Sustainable Energy Conversion and Storage. Molecules.

[4] Chen X., Wu Y., Holze R. (2023). Ag(e)ing and Degradation of Supercapacitors: Causes, Mechanisms, Models and Countermeasures. Molecules.

[5] Fares A.M., Kippke M., Rashed M., Klumpner C., Bozhko S. (2021). Development of a smart supercapacitor energy storage system for aircraft electric power systems. Energies.

[6] Guo L., Hu P., Wei H. (2023). Development of supercapacitor hybrid electric vehicle. J. Energy Storage.

[7] Zhang Y.P., Wang L.L., Zhao L.J., Wang K., Zheng Y.Q., Yuan Z.Y., Wang D.Y., Fu X.Y., Shen G.Z., Han W. (2021). Flexible self-powered integrated sensing system with 3D periodic ordered black phosphorus@ MXene thin-films. Adv. Mater..

[8] Zhan C., Lian C., Zhang Y., Thompson M.W., Xie Y., Wu J.Z., Kent P.R.C., Cummings P.T., Jiang D.E., Wesolowski D.J. (2017). Computational insights into materials and interfaces for capacitive energy storage. Adv. Sci..

[9] Lin Z., Goikolea E., Balducci A., Naoi K., Taberna P.L., Salanne M., Simon P. (2018). Materials for supercapacitors: When Li-ion battery power is not enough. Mater. Today.

[10] Ji D., Park J.M., Oh M.S., Nguyen T.L., Shin H., Kim J.S., Kim D., Park H.S., Kim J. (2022). Superstrong, superstiff, and conductive alginate hydrogels. Nat. Commun..

[11] Zhang G., Hu J., Nie Y., Zhao Y., Wang L., Li Y., Duan H. (2021). Integrating flexible ultralight 3D Ni micromesh current collector with NiCo bimetallic hydroxide for smart hybrid supercapacitors. Adv. Funct. Mater..

[12] Wu Y., Gao G., Yang H., Bi W., Liang X., Zhang Y., Zhang G., Wu G. (2015). Controlled synthesis of V_2_O_5_/MWCNT core/shell hybrid aerogels through a mixed growth and self-assembly methodology for supercapacitors with high capacitance and ultralong cycle life. J. Mater. Chem. A.

[13] Bi W., Gao G., Wu Y., Yang H., Wang J., Zhang Y., Liang X., Liu Y., Wu G. (2017). Novel three-dimensional island-chain structured V_2_O_5_/graphene/MWCNT hybrid aerogels for supercapacitors with ultralong cycle life. RSC Adv..

[14] Chen B.L., Xu L.L., Xie Z.Y., Wong W.Y. (2021). Supercapacitor electrodes based on metal-organic compounds from the first transition metal series. EcoMat.

[15] Liu Y., Qian J.L., Shi Y.X., Xu Y., Mao Y.J., Lv R.G., Huang B., Sun Y.Z., Zhao Z.Y., Chang Y.N. (2023). Latest advances of metal-organic frameworks-based materials for supercapacitors. Sustain. Mater. Technol..

[16] Sheberla D., Bachman J.C., Elias J.S., Sun C.J., Shao-Horn Y., Dinca M. (2017). Conductive MOF electrodes for stable supercapacitors with high areal capacitance. Nat. Mater..

[17] Li W.H., Ding K., Tian H.R., Yao M.S., Nath B., Deng W.H., Wang Y.B., Xu G. (2017). Conductive metal-organic framework nanowire array electrodes for high-performance solid-state supercapacitors. Adv. Funct. Mater..

[18] Shen C.H., Chuang C.H., Gu Y.J., Ho W.H., Song Y.D., Chen Y.C., Wang C.Y., Kung C.W. (2021). Cerium-based metal–organic framework nanocrystals interconnected by carbon nanotubes for boosting electrochemical capacitor performance. ACS Appl. Mater. Interfaces.

[19] Devic T., Serre C. (2014). High valence 3p and transition metal based MOFs. Chem. Soc. Rev..

[20] Han J.J., Yan Q.R., Chen Z.W., Wang Z., Chen C. (2022). Application of Cr-metal organic framework (MOF) modified polyaniline/graphene oxide materials in supercapacitors. Ionics.

[21] Yoon J.H., Jinsoo B., Cho I., Vinodh R., Pollet B.G., Babu R.S., Kim H.J. (2022). Novel supercapacitor electrode derived from one dimensional cerium hydrogen phosphate (1D-Ce (HPO_4_)_2_·xH_2_O). Molecules.

[22] Kumar R., Sahoo S., Joanni E., Singh R.K. (2022). A review on the current research on microwave processing techniques applied to graphene-based supercapacitor electrodes: An emerging approach beyond conventional heating. J. Energy Chem..

[23] Strauss V., Marsh K., Kowal M.D., El-Kady M., Kaner R.B. (2018). A simple route to porous graphene from carbon nanodots for supercapacitor applications. Adv. Mater..

[24] Kumar R., Sahoo S., Tan W.K., Kawamura G., Matsuda A., Kar K.K. (2021). Microwave-assisted thin reduced graphene oxide-cobalt oxide nanoparticles as hybrids for electrode materials in supercapacitor. J. Energy Storage.

[25] Kuang H.F., Zhang H.Q., Liu X.H., Chen Y.D., Zhang W.G., Chen H., Ling Q.D. (2022). Microwave-assisted synthesis of NiCo-LDH/graphene nanoscrolls composite for supercapacitor. Carbon.

[26] Yang X., Zhang X., Yang N., Yang L., Wang W.L., Fang X., He Q. (2023). NiCo-MOF Nanospheres Created by the Ultra-Fast Microwave Method for Use in High-Performance Supercapacitors. Molecules.

[27] Younas W., Naveed M., Cao C.B., Zhu Y.Q., Du C.L., Ma X.L., Mushtaq N., Tahir M., Naeem M. (2022). Facile one-step microwave-assisted method to synthesize nickel selenide nanosheets for high-performance hybrid supercapacitor. J. Colloid Interface Sci..

[28] Chen Y.X., Ni D., Yang X.W., Liu C.C., Yin J.L., Cai K.F. (2018). Microwave-assisted synthesis of honeycomblike hierarchical spherical Zn-doped Ni-MOF as a high-performance battery-type supercapacitor electrode material. Electrochim. Acta.

[29] He Q., He R., Zia A., Gao G.H., Liu Y.F., Neupane M., Wang M., Benedict Z. (2022). Self-Promoting Energy Storage in Balsa Wood-Converted Porous Carbon Coupled with Carbon Nanotubes. Small.

[30] Wang D.G., Liang Z.B., Gao S., Qu C., Zou R.G. (2020). Metal-organic framework-based materials for hybrid supercapacitor application. Coord. Chem. Rev..

[31] Rabani I., Karuppasamy K., Vikraman D., Ulhaq Z., Kim H.S., Seo Y.S. (2021). Hierarchical structured nano-polyhedrons of CeO_2_@ ZIF-8 composite for high performance supercapacitor applications. J. Alloys Compd..

[32] Khan U.A., Iqbal N., Noor T., Ahmad R., Ahmad A., Gao J.K., Amjad Z., Wahab A. (2021). Cerium based metal organic framework derived composite with reduced graphene oxide as efficient supercapacitor electrode. J. Energy Storage.

[33] Yuan N.C., Mei Y.X., Liu Y.W., Xie Y.T., Lin B.N., Zhou Y.H. (2022). Fabrication of UiO-66-NH_2_/Ce (HCOO)_3_ heterojunction with enhanced photocatalytic reduction of CO_2_ to CH_4_. J. CO2 Util..

[34] Sonar P.A., Sanjeevagol S.G., Manjanna J., Patake V.D., Nitin S. (2022). Electrochemical behavior of cerium (III) hydroxide thin-film electrode in aqueous and non-aqueous electrolyte for supercapacitor applications. J. Mater. Sci.-Mater.

[35] Chen K., Xue D. (2015). In-situ electrochemical route to aerogel electrode materials of graphene and hexagonal CeO_2_. J. Colloid Interface Sci..

[36] Ramachandran R., Xuan W., Zhao C., Leng X., Sun D., Luo D., Wang F. (2018). Enhanced electrochemical properties of cerium metal–organic framework based composite electrodes for high-performance supercapacitor application. RSC Adv..

[37] Hunpratub S., Chullaphan T., Chumpolkulwong S., Chanlek N., Phokha S. (2023). Characterization and electrochemical properties of Carbon/CeO_2_ composites prepared using a hydrothermal method. Mater. Chem. Phys..

[38] Singh D.L., Ghosh T.K., Mishra V., Ramasamy S., Sahoo M.K., Gangavarapu R.R. (2022). Three-dimensional lanthanide-based nanoporous metal–organic frameworks for high-performance supercapacitors. ACS Appl. Nano Mater..

[39] Chang Y.L., Tsai M.D., Shen C.H., Huang C.W., Wang Y.C., Kung C.W. (2023). Cerium-based metal–organic framework-conducting polymer nanocomposites for supercapacitors. Mater. Today Sustain..

[40] Fatemi S., Ganjali M.R., Mohammad R.G. (2022). Fabrication and comparison of composites of cerium metal-organic framework/reduced graphene oxide as the electrode in supercapacitor application. J. Energy Storage.

[41] Kumaresan L., Hanamantrao D.P., Raj S.L.S., Chenrayan S., Rangasamy B., Vediappan K. (2023). Spherically Structured Ce-Metal-Organic Frameworks with Rough Surfaces and Carbon-Coated Cerium Oxide as Potential Electrodes for Lithium Storage and Supercapacitors. Chem. Sel..

[42] Yang J., Chen L.S., Li W.W., Chen G.R., Wang L.Z., Zhao S. (2020). A novel self-supported structure of Ce-UiO-66/TNF in a redox electrolyte with high supercapacitive performance. J. Colloid Interface Sci..

[43] Dai F.N., Wang X.K., Zheng S.H., Sun J.P., Huang Z.D., Xu B., Fan L.L., Wang R.M., Sun D.F., Wu Z.S. (2021). Toward high-performance and flexible all-solid-state micro-supercapacitors: MOF bulk vs. MOF nanosheets. Chem. Eng. J..

[44] Sun T., Yue L., Wu N., Xu M., Yang W., Guo H., Yang W. (2019). Isomorphism combined with intercalation methods to construct a hybrid electrode material for high-energy storage capacitors. J. Mater. Chem. A.

[45] Du X., Zhang J., Wang H., Huang Z., Guo A., Zhao L., Niu Y., Xiang L., Wu B., Liu Y. (2020). Solid–solid interface growth of conductive metal–organic framework nanowire arrays and their supercapacitor application. Mater. Chem. Front..

[46] Yan Y., Luo Y.Q., Ma J.Y., Li B., Xue H.G., Pang H. (2018). Facile synthesis of vanadium metal-organic frameworks for high-performance supercapacitors. Small.

[47] Ojha M., Wu B., Deepa M. (2021). Cost-effective MIL-53 (Cr) metal–organic framework-based supercapacitors encompassing fast-ion (Li^+^/H^+^/Na^+^) conductors. ACS Appl. Energ. Mater..

[48] Shinde P.A., Seo Y., Lee S., Kim H., Pham Q.N., Won Y., Jun S.C. (2020). Layered manganese metal-organic framework with high specific and areal capacitance for hybrid supercapacitors. Chem. Eng. J..

[49] Liu L., Yan Y., Cai Z.H., Lin S.X., Hu X.B. (2018). Growth-oriented Fe-based MOFs synergized with graphene aerogels for high-performance supercapacitors. Interfaces.

[50] Shao D., Wang L., Lu B., Guo J., Zhang S., Lu Y. (2019). A high N content cobalt-based metal organic framework with nanorod structure for supercapacitor electrode material. J. Electroanal. Chem..

